# Supplementary material for: MetaQTL: a package of new computational methods for the meta-analysis of QTL mapping experiments
Source: BMC Bioinformatics. 2007 Feb 8;8:49. doi: 10.1186/1471-2105-8-49 (PMC1808479; doi:10.1186/1471-2105-8-49)
Supplement: Additional File 7 — MetaQTL Package : jar file and tutorial. This Zip archive contains both the MetaQTL JAR file and the files of the tutorial. [file 1471-2105-8-49-S7.zip › org.inra.metaqtl/doc/index-files/index-13.html]

N-Index


|  |  |  |
| --- | --- | --- |
| **Overview** \| Package \| Class \| Use \| **Tree** \| **Deprecated** \| **Index** \| **Help** | |  |
| **PREV LETTER**   **NEXT LETTER** | **FRAMES**    **NO FRAMES**     **All Classes** |  |


A B C D E F G H I K L M N O P Q R S T U V W X Y Z 

---


## **N**

**n** - Variable in class org.metaqtl.EMResult: The number of data points. **n** - Variable in class org.metaqtl.MultiFitResult: The number of observations without missing data. **name** - Variable in class org.metaqtl.ChromCluster: The name of the chromosome cluster. **name** - Variable in class org.metaqtl.Chromosome: The name of the chromosome. **name** - Variable in class org.metaqtl.MetaChrom: The name of the meta chrom. **name** - Variable in class org.metaqtl.Qtl: The name of the QTL. **name** - Variable in class org.metaqtl.Qtl.QTLCross: The name of the cross design. **name** - Variable in class org.metaqtl.Qtl.QTLTrait: The name of the trait. **name** - Variable in class org.thalia.bio.entity.bean.BioEntityBean: **name** - Variable in class org.thalia.bio.entity.bean.PropertyBean: **nc** - Variable in class org.metaqtl.MetaChrom: The number of chromosomes implied. **nchr** - Variable in class org.metaqtl.MetaMap: The number of meta chromosmes. **nchr** - Variable in class org.metaqtl.MetaQtlAnalysis: The number of chromosomes on which the meta-analysis has been performed. **ncs** - Variable in class org.metaqtl.CMarkerSequence: The number of common sequences. **needToBeRescaled(IBioGenome)** - Static method in class org.metaqtl.util.GeneticMapProperties: Returns true if the given map need to be rescaled - due to the mapping extansion for some pedigree designs. **NEG\_COMMON** - Static variable in class org.metaqtl.graph.ChromLayer: **NEG\_COMMON\_COLOR** - Static variable in class org.metaqtl.graph.MetaGraphPar: **newBioEntity(int)** - Static method in class org.thalia.bio.entity.BioEntity: **newCrossPopulation(int, String, IBioEntity)** - Static method in class org.thalia.bio.entity.Population: **newEstimatedLocusPosition(double, double)** - Static method in class org.thalia.bio.util.LGroupPosition: **newLocus(int)** - Static method in class org.thalia.bio.entity.Locus: Returns a new Locus from the given locus type. **newLocus(String)** - Static method in class org.thalia.bio.entity.Locus: Returns a new Locus from the given locus type. **newStaticLocusPosition(double)** - Static method in class org.thalia.bio.util.LGroupPosition: **ni** - Variable in class org.metaqtl.ChromCluster: The total number of marker intervals. **ni** - Variable in class org.metaqtl.MetaChrom: The number of marker intervals implied in this consensus. **nidx** - Variable in class org.metaqtl.TreeNode: The indices of the sub-nodes **nm** - Variable in class org.metaqtl.Chromosome: The number of markers. **nm** - Variable in class org.metaqtl.MetaChrom: The number of markers. **nmc** - Variable in class org.metaqtl.ChromCluster: Total number of common marker between clusters **nmc** - Variable in class org.metaqtl.CMarkerSequence: The number of common marker between chromosomes **nmc** - Variable in class org.metaqtl.MetaChrom: The number of marker per implied chromosome **nmqc** - Variable in class org.metaqtl.MetaQtlResult: The number of meta qtl clusterings. **nodes** - Variable in class org.metaqtl.Tree: The nodes of the tree. **np** - Variable in class org.metaqtl.QtlPartition: The number of partition. **nqtl** - Variable in class org.metaqtl.MetaQtlResult: The number of QTL **nqtl** - Variable in class org.metaqtl.QtlPartition: The number of Qtl. **NumberFormat** - Class in org.thalia.bio.util: **NumberFormat()** - Constructor for class org.thalia.bio.util.NumberFormat: **NumericalUtilities** - Class in org.metaqtl.numrec: Class Description Here **NumericalUtilities()** - Constructor for class org.metaqtl.numrec.NumericalUtilities: **NumericalUtilities.Function** - Interface in org.metaqtl.numrec: **NumericalUtilities.NRFunction** - Interface in org.metaqtl.numrec

---


|  |  |  |
| --- | --- | --- |
| **Overview** \| Package \| Class \| Use \| **Tree** \| **Deprecated** \| **Index** \| **Help** | |  |
| **PREV LETTER**   **NEXT LETTER** | **FRAMES**    **NO FRAMES**     **All Classes** |  |


A B C D E F G H I K L M N O P Q R S T U V W X Y Z 

---
